# “Talk with me”: perspectives on services for men with problem gambling and housing instability

**DOI:** 10.1186/s12913-016-1583-3

**Published:** 2016-08-02

**Authors:** Sara J. T. Guilcher, Sarah Hamilton-Wright, Wayne Skinner, Julia Woodhall-Melnik, Peter Ferentzy, Aklilu Wendaferew, Stephen W. Hwang, Flora I. Matheson

**Affiliations:** 1 Leslie Dan Faculty of Pharmacy, University of Toronto, 144 College Street, Toronto, ON M5S 3M2 Canada; 2 Centre for Urban Health Solutions, St. Michael’s Hospital, 209 Victoria Street, Toronto, ON M5B 1T8 Canada; 3 Institute for Clinical Evaluative Sciences, 155 College Street, Suite 424, Toronto, M5T 3M6 Canada; 4 Centre for Addiction and Mental Health, 33 Russell Street, Toronto, ON M5S 2S1 Canada; 5 Good Shepherd Ministries, 412 Queen Street East, Toronto, ON M5A1T3 Canada; 6 Dalla Lana School of Public Health, University of Toronto, Health Sciences Building, 155 College Street, 6th Floor, Toronto, ON M5T 3M7 Canada

**Keywords:** Housing instability, Problem gambling, Services, Men, Homeless, Complex needs

## Abstract

**Background:**

Problem gambling and homelessness are recognized as important public health concerns that significantly impact individuals, their friends and families, communities and broader society. We aimed to explore the experiences with health and social services of men who had histories of problem gambling and housing instability in Toronto, Ontario.

**Methods:**

We used a community-based participatory approach with a multi-service agency serving low-income individuals. We conducted qualitative interviews with men (*n* = 30) who had experienced problem gambling and housing instability. Our interviews employed open-ended questions to elicit men’s perceptions of services related to housing instability, problem gambling and other comorbid conditions (e.g., mental illness, substance use). We reviewed relevant themes related to experiences with services (e.g., Use of and feedback on: health and social services, housing services, justice/legal aid services, substance use services, gambling services; stigma; goals; triggers; physical health; coping strategies; finances; relationships; barriers to services and recommendations for services).

**Results:**

The concept of person-centred engagement was identified as a main overarching theme, and seemed to be lacking in most of the men’s experiences of services. Person-centred engagement for these men entailed empowerment and autonomy; empathy, compassion and sincerity; respectful communication; and tailored and holistic life plans. While there was a strong emphasis placed on independence, the men identified the importance of positive therapeutic relationships as being critical aspects of the recovery process. Based on our analyses, several recommendations were identified: 1) Increasing general awareness of services for problem gambling; 2) Delivering integrated services in a one-stop-shop; 3) Addressing mental health with psychotherapy and pharmacotherapy; 4) Providing timely access to prevention and recovery services; and 5) Enhancing life skills with peer support.

**Conclusions:**

Our study highlighted that most of the men we interviewed were not having their health and social needs met. Services need to address the intersection of problem gambling, housing instability, and other comorbidities. Ensuring services are grounded in person-centred engagement appears to be critical for optimal service delivery.

**Electronic supplementary material:**

The online version of this article (doi:10.1186/s12913-016-1583-3) contains supplementary material, which is available to authorized users.

## Background

Problem gambling and homelessness are recognized as important public health concerns significantly impacting individuals, their friends and families, communities and broader society [1–6]. Broadly, *problem gambling* refers to behaviors causing harms ranging along a continuum from moderate to extreme [7] and is defined as *“difficulties in limiting money and/or time spent on gambling which leads to adverse consequences for the gambler, others, or for the community”* [8]. On a more severe scale, pathological gambling refers to a mental disorder, defined by the American Psychiatric Association as ‘chronic and irresistible impulses to gamble’ [9]. Globally, the estimated lifetime prevalence of problem and pathological gambling in the adult population is estimated to range from 0.4 % to 3.6 % and 0.2 % to 3.5 %, respectively [10]. Emerging research suggests a link between gambling, homelessness and/or housing instability [11–13]; however there is little evidence on how best to provide services for persons with these intersecting needs [2].

Gambling is a common leisure activity and substantial revenue source for governments. In Canada, approximately $13 Billion dollars (CAD) are generated annually from adult Canadians gambled in the previous year [14]. While for most, gambling can be safely self-moderated (similar to alcohol consumption), there are individuals who are more vulnerable and susceptible to the harms associated with the behavior. In 2002, 1.2 million Canadians (5 %) had the potential to be or were problem gamblers [15]. For these vulnerable individuals, gambling may lead to persistent problematic behavior causing significant interpersonal problems with family and friends, educational or employment challenges, criminal or legal problems, health problems, as well as financial hardships and homelessness/housing instability [2, 16]. Moreover, persons with problem gambling are at risk for other comorbidities such as depression, anxiety disorders and suicidal ideation, as well as substance use problems [17, 18]. Similarly, persons who experience persistent financial instability are at high risk for both substance abuse and problem gambling [1, 19–25].

Recently, the importance of concurrently addressing problem gambling and homelessness due to the high rates of problem gambling among this vulnerable population has been raised as an issue [26, 27]. In Toronto (Ontario, Canada) our research team identified that the lifetime prevalence of problem gambling among 264 clients of a community homeless shelter agency was 10 % and that of pathological gambling was 25 % [26]. Similarly, Sharman and colleagues recently identified a prevalence rate of 11.6 % among individuals attending outreach centres for problem gambling in Central London (United Kingdom), with higher rates among men (20.8 %) compared to women (5.5 %) [27]. These two studies highlight the potential associations of poverty and housing instability with financial risk taking behaviour. One explanation is that individuals may exhibit more risky behaviour as a means to exit situations of extreme poverty [28], or alternatively gambling behavior may contribute to poverty and housing instability.

While there is growing recognition of the intersection of problem gambling, homelessness and other comorbid conditions such as substance abuse, there is still a gap in knowledge on the exact nature of the relationship as well as the temporal sequencing between these issues [29]. Most services are tailored to unique problems (e.g., substance use or problem gambling or housing instability), rather than the multiple and intersecting issues. Overall, there is a lack of evidence as to how and what type of prevention, screening, and treatment services should be provided [2]. Therefore, to address the paucity of research in this important area, we aimed to explore the experiences with health and social services of men who had histories of problem gambling, and housing instability in an urban centre, Toronto, Canada. A deeper understanding of experiences with services (or the lack of) from the perspectives of men who have sought housing services will help move this research agenda forward and inform the provision of services.

## Methods

This paper describes the health and social services (i.e., housing, social assistance, programs for substance use and problem gambling) experiences of men who have a history of gambling problems and housing instability. Our study received approval from St. Michael’s Hospital Research Ethics Board.

### Design

This paper is one of a series of papers from the *Problem Gambling and Housing Instability study*. A detailed description of the methods for this study has been previously published [30]. In brief, we conducted semi-structured, qualitative interviews with men who had experienced gambling problems and/or substance use problems, as well as housing instability. In addition to the qualitative interviews, participants completed a descriptive questionnaire that included collection of socio-demographic, mental health, substance use and gambling activity information.

Our project involved a community-based participatory approach [31, 32], which is defined as a *“systematic inquiry, with the collaboration of those affected by the issue being studied, for the purposes of education and taking action or effecting social change”* (p.1927) [32]. In particular, we actively worked collaboratively with our partner and knowledge-user, the Good Shepherd Ministries (GSM) of Toronto, throughout the overall study design, data collection, analyses and dissemination [33]. The GSM serves urban populations who experience severe and persistent poverty. We also used an integrated knowledge translation approach [33], which involves collaborating with our knowledge users (e.g., service providers, persons with lived experience) throughout our study. As aforementioned, we involved peer interviewers (people with lived experience of mental illness, addictions and/or housing instability). They conducted the qualitative interviews and actively participated in the data analysis process. During the peer interview-training period, a research staff member was present for the initial interviews. Participants scheduled for these initial interviews were informed that a second research staff member would be present for training purposes. If participants were uncomfortable with the second person being present, they had the option to be interviewed at a later date (after shadow interviews were completed).

### Setting

The GSM is a community-based organization situated in the inner city of Toronto, Canada, and provides services for men with housing instability. In addition to housing needs, many of the clients have multiple health and social needs, such as severe substance use and mental health concerns, unemployment, lower education and financial resources.

### Recruitment

In a previous study, members of our research team collaborated with the GSM to assess the prevalence of gambling problems among clients who visit the centre [25]. A total of 264 clients were screened (between March 4th and May 5th 2014) with the National Opinion Research Centre DSM Screen (NODs) and the short screener, the NODS-CLiP screening instrument [34–36]. Of the 264 clients, 116 had a history of problem gambling, with a large number of clients screened positive for lifetime at risk (8.3 %), problem (9.5 %) and pathological gambling (24.6 %) [25]. Of the 116 clients, 86 participants agreed to be contacted for future research and 30 participants did not consent to be contacted for follow up research. Among those who did not consent, but had a history of problem gambling, 13/30 (43 %) had NODS scores of pathological gambling (5+ score). Among the 82 men who consented to be followed up for future research, 48/82 (58 %) had NODS scores of pathological gambling (5+ scores).

For those men who agreed (*n* = 82), we collected personal contact information in the form of first and last name, date of birth, phone number and email address; alternate contacts included family members, service providers, friends, and the GSM staff who provided the contact number of the research team to those who were participants of the Drug and Alcohol Recovery Enrichment program (DARE). Those recruited through DARE had provided the program as a point of contact. The 82 men who agreed to be re-contacted were placed in a randomly ordered list, and participants were contacted sequentially from this list. We used telephone, email, and alternate contacts (e.g., family members) to reach out to the participants. Given the focus of our study was on phenomenal experience, we aimed for at least 30 interviews in order to reach theoretical saturation [37].

### Interviews

We hired people with lived experience of mental illness, addictions and housing instability to conduct the interviews. We chose this peer-interviewer approach to help participants feel more comfortable; for example, sharing their personal experiences with peers who had insider knowledge of the issues that affected participants. We also wanted to help to build research capacity among peer interviewers to give back to a community that we were asking to share very personal and often distressing experiences.

Prior to entering the field, peer-interviewers received intensive training on the project to maintain rigor and ensure safety. Peer-interviewers were given hands-on training with the interview guide and the descriptive questionnaire. A more detailed discussion of our engagement of peer interviewers is available elsewhere [30].

### Data collection

We developed a set of quantitative questions to ask participants for information on socio-demographic characteristics, gambling activity, depression [38], and drug-using behavior [39]. We used the Canadian Problem Gambling Severity Index to assess past year gambling problems [40, 41]. The semi-structured questionnaire that guided our qualitative interviews focused on open-ended questions to elicit men’s perceptions and experiences of the meaning and history of gambling, gambling experiences, reasons for gambling, issues of stigma related to gambling, housing history, contributors to housing instability and services accessed to help with housing, connections between gambling and other psycho-behavioral issues (mental illness, substance use disorders), help-seeking behavior, and readiness to change. The study peer-interviewers assisted with the review of the interview guide in terms of language appropriateness and question intent effectiveness.

We piloted five interviews in October 2013 to provide shadow training for our peer-interviewers and to refine the interview guide to improve flow and language. The remaining interviews were conducted between November 2013 and February 2014. The pilot interviews resulted in changes to the order of interview instruments (descriptive questionnaire followed by the qualitative interview); the addition of interview questions focusing on specific service utilization patterns. Each participant completed a single interview. The interviews ranged in time between 30 and 90 min.

Of the 82 men who had consented to be contacted for future research from the previous study [25], we completed 30 interviews (including the five pilot interviews). Notably, we were unable to reach 52 men for various reasons: phone number or email not working, repeatedly missed scheduled interviews, refused, or were found to be ineligible to participate. Of the 30 completed interviews, the majority of the interviews (*n* = 22) were conducted onsite at the GSM and the remainder was conducted at St. Michael’s Hospital. Participants received $50 in compensation for participation. Given the sensitive nature of the information being collected, we only required verbal consent for the interview and for audio recording of the interview. Audio files were transcribed verbatim.

### Analysis

Data coding and analysis occurred simultaneously with data collection, using Grounded Theory [42], among seven research team members (CP, FIM, KD, SHW, SJTG, RB, JWM). Our coding methodology followed that proposed by Burnard [43]. Open coding was used with seven interviews. We selected these interviews based on the following criteria: a range of different time points in the data collection process (one pilot and six post-pilot); a range of representative interviews from each of the peer interviewers; and, a range of participant scores on the NODS. Each team member (CP, FIM, KD, SHW, SJTG, RB, JWM) was assigned two of the seven transcripts to review to ensure that coding between team members was consistent. After reviewing the assigned transcripts, we met to discuss the initial macro and micro codes; and to develop and agree upon macro and micro (sub) theme names and their descriptions. Defining the themes of coded material and providing specific examples within a theme ensured that coders understood the meaning of each theme for later coding activity. All coders were involved in the development of the codes. As a result, there was minimal disagreement in the codes and their definitions; any discrepancies were discussed among team members to achieve consensus. We met with the peer interviewers to discuss the findings and confirm accuracy of interpretations.

We next conducted focused coding (using NVivo 9.2) wherein each team member independently coded a selection of 11 transcripts, including the seven previously used in open coding. As new codes emerged, these were integrated with the initial codes list and formed the basis for coding the full set of 30 interviews. While coding, we focused on identifying ambiguities in the names of the themes and their descriptions; tips/hints (coding rules) to differentiate between similar codes; potential missing themes to be added to the codebook; and areas of text difficult to code so that the team could come to a consensus on where best to place the information.

New emergent themes were continuously integrated with the initial codes and previously coded interviews were re-analyzed with these new codes. This method ensured that the codes were broadly representative of all interview data without duplication of themes. After all coding was completed, the team assessed saturation of ideas among the themes in the interview guide and the research objectives. For this particular paper, a smaller group of team members (SJTG, SHW and WS) reviewed relevant nodes/themes related to experiences with health and social services, facilitators and barriers, and recommendations for services. In particular, themes previously identified in the coding framework development process (Use of and feedback on: health and social services, housing services, justice/legal aid services, substance use services, gambling services; stigma; goals; triggers; physical health, coping strategies; finances; relationships; barriers to services and recommendations for services) were reviewed for sub-themes as they related to experiences with health and social services. The team members met to discuss the relevant themes and interpretation of findings. In presenting our findings, we have replaced the participant identifying numbers with pseudonyms. We took the last letter of the participant’s name, and chose a first name that started with that letter, with a an explicit effort to select a first name that was consistent with the participants’ ethnicity.

## Results

The men we interviewed (*n* = 30) ranged in age from 26 to 77 years (mean age of 48 years). Most of the men were not in a current relationship (*n* = 19, 63 %), had an education of high school or equivalent (*n* = 21, 70 %) and were unemployed at the time of the interview (*n* = 24, 80 %). Based on the scoring algorithm for the Canadian Problem Gambling Severity Index, 77 % of the men (*n* = 23) in the sample met the criteria for problem gambling with negative consequences, 13 % with moderate problem gambling (*n* = 4) and 10 % with low level or no problem gambling in the past year (*n* = 3). The main source of income for the men was through government assistance such as social assistance (*n* = 7), employment insurance (*n* = 6), and disability support program (*n* = 10). The likelihood of having a mental disorder (psychological distress) among the sample of men in the 4 weeks prior to the interview was high, with 83 % (*n* = 25) of the sample presenting as having mild to severe psychological distress (anxiety and depressive symptoms) based on the Kessler Psychological Distress Scale [38, 44].

### Person-centred engagement

**Fig. 1:**
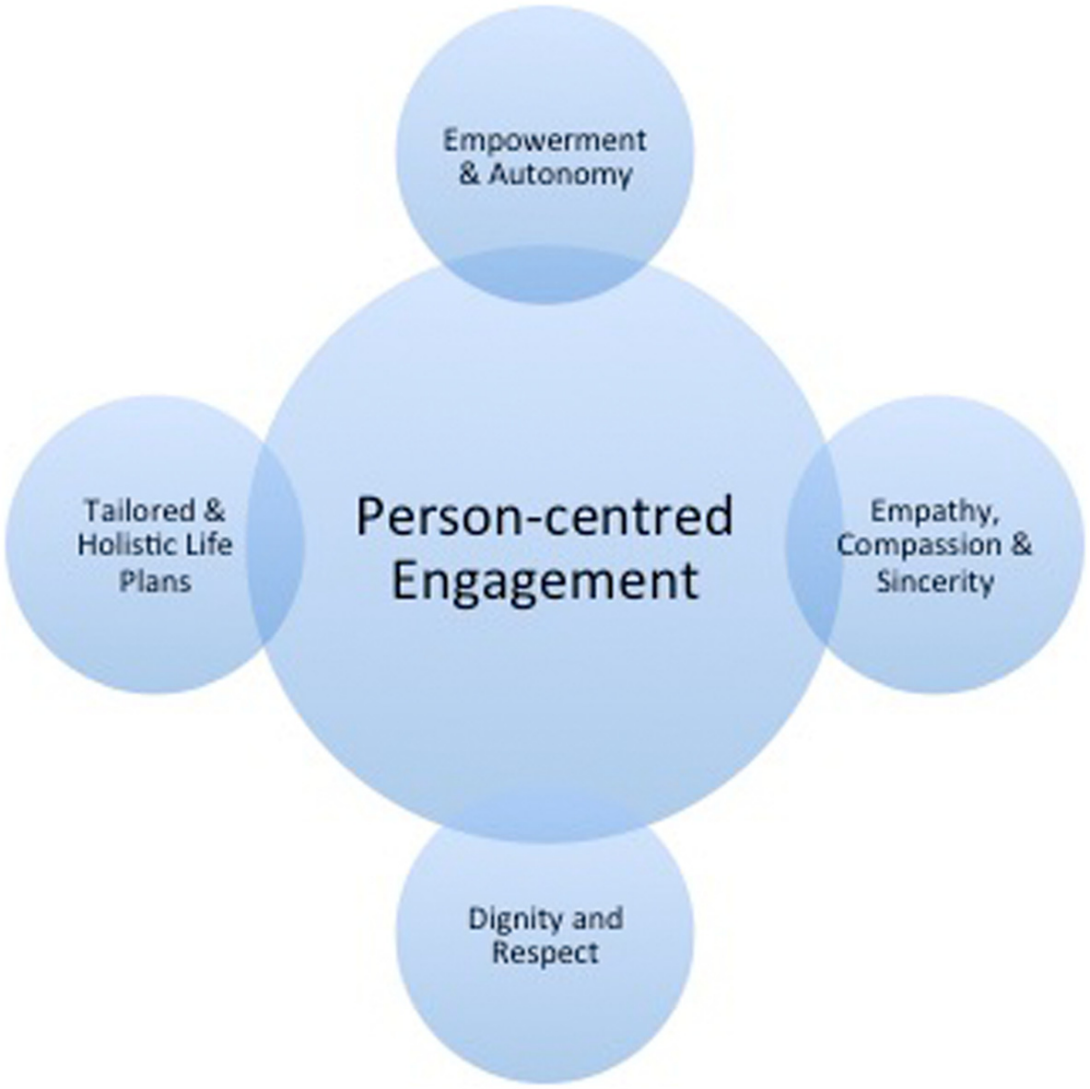
Key components identified of person-centred engagement

The concept of *person-centred engagement* was identified as a main overarching theme. Key components of person-centred engagement for these men were comprised of the following (see Fig. 1): empowerment and autonomy; empathy, compassion and sincerity; respectful communication; and tailored and holistic life plans. In the following section we outline the components of person-centred engagement identified in our data.

#### Empowerment and autonomy

Many men described their personal inner strength and ability to take ownership of their situations as an important driver of their journey to recovery. Participants reflected on the loss of autonomy with their gambling behavior, and their attempts to regain control with various strategies. The men talked about self-imposed parameters or self-management strategies they put in place to limit access to funds (e.g., money jar for gambling) or to gambling venues. Others spoke about reducing behavior progressively; that is, visiting a venue every day to a few days per week, or gambling less frequently and ‘more responsibly’. Sometimes services were not desired or not perceived as helpful based on previous experiences with service providers. One of the aspects that made services less helpful was the perception of being ‘told what to do’ and not feeling empowered.

**Geoff:***No. Because they [service providers] tell you what to do. I used to go every day. Now once every week, maybe three, four, five times a month. Cut down, but big money. So, was no good. You have to be strong willed. You have to have a strong will power.*

The men spoke about the realization that they knew they had to do this themselves, to have ‘will power’ and autonomy. Many were still in the process of ‘working on themselves’ and spoke about the investment they had made, both financial and through personal time.

**Eduardo:***So, it took me a while to work on myself to get myself to where I am today. I’ve worked on life. I spent, like I said, I’ve spent a lot of money on myself, and I’m still working on myself.*

There was a sense of pride in the ability to be autonomous, self-recover and not rely on services, as reflected in Mark’s comment:

**Mark:***I’ve never gone to a housing worker. I’ve had housing workers here, like at the [agency], but even these guys, like they always have said that they look up to me because I’m the type of person that will come into the [service agency], and pound the pavement to look for a place, and within 2 to 3 weeks, like I have a place.*

We identified a tension between providing the necessary supports for the men while also respecting their need to self-manage, and feel empowered, as well as gain the necessary skills for future independence. This tension was particularly apparent with respect to differing views on what are the best delivery models of financial support for persons with problem gambling. For example, one model proposed by a participant was to deliver automatic payments for housing and groceries from social assistance in order to ensure that essential bills would be covered. Shane’s quote below reflects this tension of how much support is appropriate, with his view that certain people require more structured guidance with finances and less autonomy in the short term.

**Shane:***… need today, to stop thinking about people’s dignity when it comes to that situation [gambling Ontario Works money away]. Hold them by the hand if you have to. Take them to the landlord, okay, let’s sign, and I want you to sign the form for auto pay, and then we’ll take it back, or from the office, and take the landlord, and here’s your cheque. Let’s put it on auto pay. Forget about saying, I’m more worried about your human rights, or how do you feel... That doesn’t cut crap with people. How much money they’re throwing away…ODSP [Ontario Disability Support Program], they grab a $1,000 cheque and the next day they’re done. They’re in [service agency], what happened? I gambled it all away, or I smoked crack all night, and I’m broke.*

#### Empathy, compassion and sincerity

While there was a strong emphasis placed on independence, the men identified the importance of positive therapeutic relationships as critical to the recovery process. Several men described previous negative experiences with various providers across health and social sectors (e.g., housing), which had longstanding impacts on as to whether or not they later reached out for care. In addition, several men expressed a feeling of embarrassment and weakness as exemplified by Yuri’s comment:

**Yuri:***Like I said with gambling or drinking, whatever, but just speaking to a regular doctor, I thought this is not their specialty. I felt somewhat embarrassed because I looked at it like…a sign of weakness, and kind of like not mentally stable, and I was worried about coming across that way. I didn’t want to feel that way. I especially more recently being in an environment of shelters, I seen people with mental health problems, I didn’t want to be like them. I didn’t want to be associated like as being someone like that.*

There was also a fear of stigma and judgement from society due to the negative impact problem gambling may have on various aspects of one’s life such as housing stability and interpersonal relationships. Spencer’s comment highlights this fear of stigma and judgment, as problem gambling can affect many aspects of one’s life (e.g., housing stability, personal relationships, financial stability):

**Spencer:***I just think it’s none of anybody’s business except my own because society looks down upon people, I find. People with gambling, when it comes to, I mean a lot of people lose their house. The marriage is gone, the car is gone. Society can look down on that as gambling as a bad thing…*

The men also mentioned that there was a disconnection between key messages delivered in treatment programs versus the reality experienced. Geoff’s comment below reflects this disconnect:

**Geoff:***Well, they got what you call 12 steps, and they got 12 traditions, and one of the 12 traditions is that you don’t judge other people, yet wherever I went people judged me.*

Given these vulnerabilities for disclosure and apprehensions about being identified with others they see as having addiction and mental health problems, the men highlighted the need for empathy, compassion and sincerity during interactions with service providers.

**Mark:***So, it’s…have more compassion for people that drink and do drugs and gamble. Try to help them, and get more programs out there for people with addictions and gambling. Like get more stabilized housing for them. Get more counsellors for them, you know.*

**Todd:***You can tell if somebody’s in it for a pay cheque, or they’re in it to make a difference because they’re in it to make a difference. The others, I only got to know some of them, but I see a lot of them as more of a pay cheque thing.*

#### Respectful communication

Most men were concerned with feeling valued and respected while interacting with services or service providers. The men often talked of low self-esteem and self-judgment of their own behaviours, which is the other side of the self-confidence in self-management described earlier. They were acutely aware of the ways that others, especially professionals, communicated with them; which could include perceptions related to not being trustworthy. In the following scenario, the participant Todd describes experiencing prejudice in one pharmacy, where he was refused medication for a prescription and felt misjudged as being only there to get drugs to sell. Conversely, Todd describes a more client-centred response that he eventually received at another location, which led to re-evaluation of his medications and a prescription with fewer side effects.

**Todd:***The situation was the pharmacy judged about…thought I was wanting Percocet’s to sell for drugs, that wasn’t the case. I wanted Percocet because I was in pain. Now thank goodness, I come down here, got re-evaluated, now it’s not Percocet’s, I’m on sulphate, morphine, it works a lot better. It’s not as damaging…*

For the men, respectful communication is not presumptive, suspicious or judgemental. Rather, it affirms the dignity of the client so that they feel they are genuinely being care for by the health providers, as demonstrated by one of the men’s experiences:

**Mark:***My family doctor, and the counsellor that he’s got me connected to are very helpful. Like, they don’t put me down. I go into my doctor’s office, and I’ll be all fucked up on crack and he’ll go, go see the counsellor. So, I’ll go see my counsellor. They never, ever put me down. Like, they never say, you’re an asshole, or you’re a crack head, you’re a big alcoholic, you need to get help. They never do that. They try to ease me off it.*

It was evident from the interviews that these men lacked supportive informal social networks with family and friends, which may increase the value placed on active listening by (or on the part of) service providers.

**Yuri*:****…at least someone who says, ‘what’s on your mind, let’s talk about it,’ I think that’s important…. I think just give you the opportunity to talk.*

Several men highlighted the importance of respectful communication, which inclined them to engage with the service provider. In the absence of such communication, they became not simply disengaged, but antagonized.

**Todd:***… [If] I think they’re talking to me not with me, they’re talking at me, not with me, I’ll stop communication right away. I’ll say, look it, you’re talking at me, talk to me, talk with me, but don’t talk at me, don’t talk down to me.*

#### Tailored and holistic life plans

The men spoke of the need to have tailored and holistic plans that addressed goals specific to their situations. These men saw treatment and recovery as a comprehensive process that needed to include physical and mental health, education, employment and housing.

**Shane:***Getting more personal, one-on-one with the clients. You know, they’re very detached nowadays, and I guess it’s a sense of jadedness knowing that…we know what you’re doing with your money, but we’re not going to, you won’t tell, we won’t tell, and no harm, no foul whereas they need to be more one-on-one, what do you need…What do you need, do you need a program, school, that kind of thing. Being able to know a person’s personal file. Like my OW [Ontario Works provider] knows me very well. Knows exactly what I need and what I can’t get because of it. That kind of thing… They need to be more one-on-one with the people.*

Most of the men described experiencing mental and physical disabilities, which affected their ability to participate in society (e.g., maintain meaningful employment, healthy relationships). The men identified the need for individualized services that also address the multifactorial issues that may need to be addressed, related to areas such as interpersonal relationships, mental and physical health, housing, financial management, education and employment.

**Oscar:***I suffer from bipolar. So, for me every day is a struggle, you know. Lately I been drinking more than I used to, you know. Of course I have free time. I have nothing else to do…*

Many of the men described not just an illness or injury, but a process that triggered a downhill spiral of problem gambling, housing instability, other addictions, and losing relationships as a consequence.

**Laurent:***I got cancer. I’m an asthmatic, I have been since birth, and I have osteoarthritis, degenerative disk disease. I can no longer work, my wife went to work, I became a stay at home dad, and then, my wife couldn’t take it because I was prescribed opiate, and methadone, and all kinds of pain killers, and she couldn’t take it anymore, so we got separated, and I lost my house… I been separated about 10 years now. I lost everything that I had. I had rental homes, I used to flip homes, and I lost everything because I got ill. I haven’t seen my kids for 10 years.*

### Recommendation for services

In reflecting on their experiences, the men had insightful reflections on the services received (or not received to date), and provided numerous suggestions for future services. The following section will summarize the key components of these service delivery recommendations:

1. *Increasing general awareness of services for problem gambling*
  There seemed to be a lack of awareness of the interplay between problem gambling and other addictions among some of the men we interviewed as well as their care providers. While the men might have been told they had problems with gambling by their health care provider, most men reflected that they were not told they could get help or where to go for treatment and likely would have benefitted from enhanced system navigation support.
  **Spencer:***So, he [physician] just basically said try not to gamble, he didn’t tell me where to go to get help or anything like that because it was such a brief…I know it was brought up, but it was so brief. Like he said that it would increase my anxiety, and it’s not a big relationship question, but I don’t know.*
  **Oscar:***I don’t know. Maybe if I knew there was help out there, I would have asked, you know. I didn’t know that people get help for gambling. Like now, you show me the list [resource list from study team]. I didn’t know. People say you got a problem with alcohol, you got a problem with drugs.*
2. *Delivering integrated services in a one-stop-shop accessible environment*
  The majority of the men we interviewed identified an overall challenge in obtaining services to address their complex and multiple needs (e.g., housing, gambling, substance use, disability, employment). Treatment services seemed fragmented and disconnected from each other.
  **Shane:***There is no program that deals with addictions and gambling in a mixture. It’s either one or the other. You get gambling or you do substance abuse first, and then, you go to gambling, that would be a good idea.*
  Several of the men described the idea of having a physical “*one-stop-shop”* to address their multiple health and social concerns. Integrating services into one physical location would assist the men with transportation challenges, as many participants described the financial challenges and the time required using transit to access different services across the city.
  **Mark:***…It’s one simple thing. Have a mega treatment centre with counsellors and social workers. Instead of saying, okay I’m a social worker. Okay, I’m going to send you to this counsellor. You go to this counsellor, this counsellor goes, okay, I’m going to send you to this detox. This detox says, I’m going to send you to this treatment centre. This treatment centre goes, okay I’m going to send you back to [service agency] so that they can get you housing. Why not have it all under one building and save tax payers a hell of a lot of money, and your OHIP [Ontario Health Insurance Plan] card a hell of a lot of money…*
  Balanced against the importance of having a one-stop-shop, several men reinforced the need for services to be tailored to their unique needs. For example some men may have challenges with gambling and other addictions, while others may not. Services need to reflect this heterogeneity.
  **Tom:***…I mean, it might sound obvious to say, you know, somebody who’s drinking is more likely to gamble, and can be a worse gambler because obviously drinking impairs the judgment. But I met people at the casinos who were always sober and clearly had a gambling problem, and I’ve met people who drink…I’ve met people who just have one, just drinking, or just gambling, and me I had the combination. So, you know, clearly there’s a connection there that’s common in some people and not in others.*
  Locating services in a ‘safe’ area was also identified as an important concern. Several of the men identified that services are physically located in areas that expose them to triggers (e.g., drug dealers). Elliot described how placing a rehabilitation facility in an area that has high rates of illicit drug use decreases the likelihood of successful rehabilitation.
  **Elliot:***This [service agency] is in the middle of [name of park]. It’s the biggest crack haven in the entire city, okay. So, let me get this straight. We’re smart, we’re going to put a rehab right in the middle of crack land. Are you kidding me. What do you think the success rate is there? It’s horrible. You can’t even go to the store and get a pack of cigarettes without some dealer in your face. Right, this is a problem. This needs to change.*
  The physical layout and interior design of treatment services was also identified as an important element of service delivery. Shane reflected that physical layout and provider attire (e.g., lab coats) impacted the extent to which he felt compassion. Shane preferred service providers wear their own clothes rather than gowns that reflected the idea of a hospital.
  **Shane:***Detached clinical atmosphere. It was like a mental hospital, it had that feeling it was a mental hospital. The staff were there to collect their pay cheque. They didn’t really have any compassion. It was very cold, and it was like detached…they were all in the hospital, like the personal support gowns and stuff like that. It was like very cold and clinical whereas the [name of treatment centre], they had their own clothes. You were allowed to talk to them in their office at anytime.*
3. *Addressing mental health with psychotherapy and pharmacotherapy*
  Mental health was a common concern for the majority of participants we interviewed. The men expressed a common need to be heard and to explore the roots causes of their respective mental health challenges. Moreover, many men felt frustrated by the inability to access publicly-funded counselling services (e.g., psychotherapy) so they might be able to address their mental health concerns over the long-term. Most of the men who received mental health services were treated by psychiatrists and expressed limitations in not being able to discuss their situation to the extent they had wanted. Specifically, the majority of men acknowledged the need for comprehensive multi-disciplinary treatments (e.g., pharmacotherapy integrated with in-depth psychotherapy).
  **Elliott:***I think the big fault with our health system is listen I know I have a drug problem, and you can go to meetings and you can go to rehabs, and they’re all great, they’re still not telling me why… So, at the end of the day, the crack’s not the problem, the gambling’s not the problem, that’s the solution to us. That’s our solution, right. So, we have to figure out what’s causing the problem in the first place. We’re not doing that…*
  In addition to receiving more psychotherapy, several men expressed a need for improved pharmacotherapy, such as increasing access to medications (i.e., obtaining prescriptions from physicians and getting medications filled). In particular, access to prescription medicine was identified as a major challenge when a person does not even have access to a regular family physician, financial means to pay for the medication prescribed, and appropriate identification to qualify for eligible services.
  **Oscar:***No drug card (Ontario Health Insurance Plan card). I was actually to be honest with you, I lost all my IDs. I lost just about everything, and I didn’t really care, and I was like, so what I lost my ID. So, I’m homeless, what do I want my ID for. I was lost. I wasn’t really thinking that my…that I was sick. I wasn’t taking the medication, and not taking the medication, the sickness took over [bipolar disorder]. I got into a lot of trouble, and I was how you say? I was living in a different world. Different reality. That’s what my doctor said.*
  One man described losing his disability benefits because of a paperwork problem and consequently being unable to take his anti-depressant medication.
  **Yuri:***… I’m on medication now, I know that it will contribute to the fact that I don’t feel as depressed as I normally could feel, but I went through a period of time when I wasn’t taking medication, and that was a real rough patch. That was about 4 months ago when I went about a month without medication only because the paperwork within ODSP [Ontario Disability Support Program] got fouled up and I wasn’t getting my benefits, and just arguing with them.*
  Taking medication as prescribed was also noted as a significant challenge due to the various side effects (e.g., drowsiness). These side effects were noted to not only affect the men’s interpersonal relationships but also pose risk to employment status and their housing stability.
  **Oscar:***… Yeah. I over reacted. I was skipping my medication, and I was really getting really bad. No sleep, and because I had to get up so early to work, and I told the guy there if I take my medication I cannot get up in the morning to work. I decided not to take the medication, and I wasn’t taking the medication to go to work because you have to work, to stay in the program you have to work. So, for me to get out at 6 o’clock in the morning I couldn’t take the medication because the medication, the medication I take, it doesn’t allow me to get up that early. No. I had a problem with one of the guys and I got kicked out. I was kind of angry with them because I told them sometimes if I don’t have the medication I may say things, little things that I don’t normally I would do, you know. They never listened to that. So, they kicked me out. I been back there only to see my doctor.*
4. *Providing timely access to prevention and recovery services*
  Several of the men identified the need for improved timely access to services, as often the men were in crises and required urgent help. The wait-times to get into rehabilitation services for substance use were identified as problematic (e.g., several months). Elliott describes below calling services for help with his drug addiction, only to be told that the wait time was several months:
  **Elliott:***…you got to wait 2 to 3 months. Do you know what can happen to somebody in 2 to 3 months, right… These things are set up for people to fail…*
  *…I know that it’s a snowball effect. When you’re in that way of living, it snowballs, and when that ball’s rolling down that hill, you ain't stopping it. So, when you’re saying at the end of the process right before you’re about to get homeless, forget it, it’s too late.*
5. *Enhancing life skills with peer support*
  Several of the men we interviewed identified the need for enhanced training in basic life skills, such as learning important self-management strategies. Many of these men were raised in abusive home environments during their formative years and had not learned important daily life skills such as budgeting, and stress and anger management.
  **Taylor:***Maybe just like yoga studio or something like that, or try to release from all the stress that you’re going through your body, and what not, and just focus, and relax yourself to a certain state that… and be peaceful to your body, mind, and spirit for the most part.*
  Further, many men described the value in peer mentoring by others with similar experiences, and learning from their lived experiences. Many men described losing family and friends due to their addictions, and there was an expressed need for informal support.
  **Yuri:***At that time, yeah. I thought if I could just meet somebody who is going through it, has gone through it just to talk to them, they don’t even have to be professional, just someone like you [peer interviewer] for example. If I had met you back then, I would have thought this is great because I can beat it. If he beat it, I can beat it, you know. Like I said, I see the light at the end of the tunnel. Thinking all this crap that I’ve gone through for like the last 10 years, I can get out of it, slowly, baby steps first.*

## Discussion

We explored the experiences with health and social services of men who had a history of problem gambling, and housing instability in an urban centre. The *Problem Gambling and Housing Instability Study* is one of the few studies to our knowledge that examines the intersection of needs for men with problem gambling and housing instability. Given the current knowledge gap about these complex and often interrelated issues [18, 45], our results offer several important contributions to the literature. Our findings highlight the importance of person-centred engagement in service delivery, which for these men included: empowerment and autonomy; empathy, compassion and sincerity; respectful communication; and tailored and holistic life plans. These characteristics of person-centred engagement are similar to the growing body of literature in health care more generally [46–52] and specifically for those with complex health and social needs [18, 53–55].

### Providing culturally-sensitive and communication training to providers

Based on our qualitative data, there appear to be important areas for improvement, not only with the types of services provided, but also in the manner in which they are delivered. The men described ongoing challenges with perceived stigma and judgment, which has been previously identified as a barrier in seeking care for this vulnerable population [56–58]. Improving communication competency by providing training for service providers may minimize the stigma and judgment that the men described experiencing in their interactions with providers, such as being open-minded and not having preconceived assumptions [59]. The provider-patient relationship has been identified as important to treatment success for persons with mental health problems [55]. Notably, positive therapeutic communication, alliances, and the active involvement of patients in their treatment planning were identified as being beneficial in mental health care [55], and presumably would also be important for persons with problem gambling. As our study identified, feeling autonomous and directing their care plan and overall life were identified as important components for the men we interviewed.

### Increasing awareness among service providers on problem gambling and comorbid conditions

Additionally, our findings suggest the need for increasing awareness among service providers and the general public on the intersection of problem gambling and co-occurrence of housing instability or health comorbidities. In reflecting upon the type of treatment received, most men noted they received treatments for their substance use problems or services for housing instability, but did not receive formal services for problem gambling. Previous research has identified that persons with problem gambling often seek treatment for other comorbid conditions (e.g., depression or substance use), and have low self-awareness of their problem gambling [18, 25, 56], which places more importance on the role of providers to screen for problematic behaviors. Similarly, Dowling and colleagues recent systematic review identified the need to improve routine screening and assessment of problem gambling and psychiatric co-morbidity [18]. Specifically, the review found that 75 % of persons who seek treatment for problem gambling have co-morbid Axis I disorders, with the most common conditions being nicotine dependence, major depression, alcohol abuse and dependence, social phobia, generalized anxiety disorder, and post-traumatic stress disorder. Therefore, providing more education and training to service providers on the importance of screening for problem gambling, and availability of services would be helpful in optimizing referral pathways and the uptake of needed services [25, 60].

### Increasing the availability of and the type of integrated services

Moreover, there is a need to increase the availability of and the type of integrated services for persons with problem gambling. Current programs such as Gamblers Anonymous address problem gambling but may be ineffective at addressing the housing instability, comorbid substance use disorders and mental illness often experienced among individuals with problem gambling issues [61]. Our findings reinforce the importance of integrated and tailored care related to medical, housing and psychological care in a comprehensive one-stop-shop physical setting [18, 45]. Specifically, integrated care would involve one care plan among a multi-disciplinary team (which includes the person experiencing problem gambling) that addresses multiple complex health and social needs [62]. Importantly, most of the men we interviewed expressed a desire for intensive psychotherapy to understand the root causes of why they engage in certain behaviours and to receive ongoing help with their mental health and addictions. Providing comprehensive psychological services in an integrated facility would be of value to address the intersection of these complex needs. Our findings are supported by recent work by Cowlishaw et al., who found that persons experiencing problem gambling were 8.5 times more likely to use psychological services compared to those with no gambling problems [2]. In addition, their study showed that persons with problem gambling had significantly more anxiety, panic disorders, phobias, somatic symptoms, fatigue, concentration difficulties, sleep problems, irritability, suicide ideation, history of suicide attempts, and acute inpatient visits compared to those with no gambling problems. Indeed, providing integrated and individualized services may be more cost-effective compared to the current models of service delivery, which seem to be more crises driven, rigidly structured and fragmented [63].

### Providing a ‘stepped-care approach’

While not specific to problem gambling, a report by the Ministry of Health in British Columbia reviewed the evidence on different types of integrated models of primary care and mental health and substance use services in the community [62]. Of note, similar barriers to care were outlined in this report as we found in our study, such as issues surrounding stigma, access, unmet medical needs, and lack of training and knowledge [62]. Importantly, this report highlighted the importance of *‘stepped-care’* approach, which involves the least intrusive and most cost efficient treatment for an individual with the ability to adjust based on individual’s (episodic) needs [64]. The models of care proposed in the report range from *communication approaches* (addressing mild to moderate severity of needs) to *integrated team approaches* in one physical space, which wrap services around the individual (addressing severe and persistent complex needs). Our study reinforces the importance of co-locating services in one physical space and providing a welcoming person-centred environment [64]. Positioning clinics in close proximity to shelters might optimize use of services [53, 56]. Finally, most men we interviewed seemed to lack informal support systems that might be useful to facilitate system navigation. Previous literature has shown that persons with problem gambling have smaller social networks [2], and there might be benefits of peer mentoring within the integrated team models to assist with self-management strategies.

Importantly, our study identified an apparent tension that exists between providing the necessary supports for health and social well-being, while also respecting and maintaining autonomy. Several of the men noted a need for more structured support and guidance with their finances in order to ensure that essential bills were paid (e.g., housing and food). In the absence of this support, the men reflected that the financial assistance payments would be spent on gambling and other addictions. A ‘stepped-care’ and tailored approach with the active engagement of the individual might allow for tailoring the type and extent of support (e.g., financial management) to particular needs.

### Limitations

There are several limitations to the study that should be acknowledged. Firstly, we only interviewed 30 men who had lived experience with problem gambling and housing instability. These findings reflect their experiences and are not necessarily generalizable to a broader context or the general population; however our intent was specifically to talk with experts (those with lived experience) to gain in-depth knowledge of the needs of men who experience problem gambling. Secondly, we have not included the perspectives of women, service providers or decision-makers in this study; however, we recognize the importance of capturing these perspectives and our team is currently writing a paper to capture these viewpoints. Thirdly, these findings are from the perspectives of men who live in the Greater Toronto Area, however they reflect the context of a multicultural urban environment and might be useful to inform service delivery other urban settings. It would be important to extend future work into other geographical settings and among women to compare and contrast the differences and similarities in service needs and delivery models.

## Conclusions

Our study highlighted how most of the men we interviewed were not having their health and social needs met within the current delivery models in Toronto. Services should address the intersection of problem gambling, housing instability, and other comorbidities. Person-centred engagement was identified as a key overarching theme in our work. Future research is needed to examine whether the components of person-centred engagement are similar for other men in different geographical settings, as well as women who are experiencing problem gambling and housing instability. Ensuring services are grounded in person-centred engagement seems to be critical for optimal service delivery. Additionally, future research is warranted to explore the perspectives of service providers and decision-makers (our research team has a paper in process exploring the perspective of service providers), and how might the models identified for mental health and substance use problems be adapted to meet the needs for persons with problem gambling and housing instability. Finally, we are currently exploring the type of education and training required for providers to improve person-centred engagement and the quality of services provided for this vulnerable population. Integrating these different perspectives of key stakeholders will help inform the development of interventions addressing the intersection of these complex needs.

## Abbreviations

DARE, Drug and Alcohol Recovery Enrichment Program; GSM, Good Shepherd Ministries; NODS, National Opinion Research Centre DSM Screen for Gambling Problems

## Additional file

Additional file 1: Interview guide (DOC 53 kb)
