# Supplementary material for: “Talk with me”: perspectives on services for men with problem gambling and housing instability
Source: BMC Health Serv Res. 2016 Aug 2;16:340. doi: 10.1186/s12913-016-1583-3 (PMC4971622; doi:10.1186/s12913-016-1583-3)
Supplement: Additional file 1: — Interview guide (DOC 53 kb) [file 12913_2016_1583_MOESM1_ESM.doc]

**Interview Guide (Condensed Version)**

# Introduction

# ***You have been invited to share your experiences with housing instability and gambling. Thank you for agreeing to participate. I mentioned in the consent letter that the interview should take 60 to 75 minutes. We do appreciate the time you’re giving us today and don’t want to keep you here longer than 75 minutes so we’ll do what we can to stay within the time limit.***

Why don’t we start with an introduction? Could you please tell me a bit about yourself?

Now I want to ask you some questions about your gambling experiences.

Could you begin by telling me what ‘gambling’ means to you? Please tell me about your own history with gambling. What are (were) your reasons for gambling?

Can you tell me anything more about what gambling does/did for you? Please tell me how gambling makes you feel.

Now I want to talk to you about your experiences of gambling and how it relates to other things in your life: for example to drug use and drinking.

Do you (did you ever) have problems related to alcohol and/or drugs?

If ‘yes’, can you describe them?

How do drug or alcohol use and gambling interact for you? For example, does one sometimes lead to the other? Please tell me about how you (used to) acquire the money you need to gamble?

Do you, or did you ever, consider yourself addicted to gambling?

When did you feel gambling was becoming a problem?

Why did you feel this way?

Did you ever seek professional help for gambling problems? Who did you seek help from or where did you go? Please describe those experiences for me – positive ones and negative ones.

Did you ever seek support from non-professionals like family, friends, clergy members? Please tell me about this.

When you did seek help for gambling problems, what was your housing situation like? Tell me about that.

Have you ever conned anybody to get money? (e.g. 3-card monte, pool, poker hustling) What did you do? Can you tell me about that?

Do you think that gambling has affected your stress level and anxiety in anyway? Have you ever felt panicked due to your gambling? Have you ever felt depressed? Can you describe this please?

At any point in your life, have you looked for help to quit or cut down your alcohol or drug use? If so, please tell me what went on and how it worked out.

Have you ever tried to get help or has anyone ever tried to help you with your gambling and alcohol/problems at the same time? Can you tell me about that?

[***Interviewer: If NO, please ask the following question***].

Did you ever wish you could find treatment to deal with these issues at the same time? What types of services would have helped?

What wasn’t helpful?

What types of services would have helped?

What was missing?

[***Interviewer: If no to the question above, please ask, If YES, please skip to the next question***].

What services would have helped you and could help others who have gambling problems or substance use and are facing <please refer to current living situation or ‘housing situation’> /homelessness?

I’m going to read from a list of services that may help men at risk of problem gambling or other problems, like emotional distress and substance use problems. For each type of service, please let me know whether you are familiar with or whether you have used or had experience with such services. Then I’m going to ask you whether you think the services are useful and how they could be improved:

a) Talking to a psychologist, psychiatrist or counsellor

b) Social workers

c) A shelter or other housing service

d) Legal aid

e) Substance use or addictions support

i) Can you think of other services I may have missed? __________________

[***Interviewer: for each service the respondent used, please probe each by asking:

Do you think such services are useful, how could they be improved?

Are there services that should be available but are not for men experiencing gambling or other problems?

Do you think your housing would be stable if you had not been/were not gambling?

Has/would getting housing helped you with your gambling?

Has/would getting housing helped you with your alcohol and/or drug use?

Is there anything else you would like to add to our discussion? Is there something we didn’t ask that you think would be useful to understand gambling and <please refer to current living situation or ‘housing situation’>?
